# Supplementary material for: Is autumn the key for dengue epidemics in non endemic regions? The case of Argentina
Source: PeerJ. 2018 Jul 17;6:e5196. doi: 10.7717/peerj.5196 (PMC6054063; doi:10.7717/peerj.5196)
Supplement: File S2 [file peerj-06-5196-s002.docx]

Temperature dependent model of the extrisinc incubation period (EIP)

The proportion of the EIP completed daily was calculated using a model developed by Jetten & Focks (1997) modified to estimate the completion of EIP in each hour:

| $\text{r}\left(\text{T}_{\text{h}}\right)=\left(\text{p}_{\left(25^{\circ}\text{C}\right)}\left(\text{T}_{\text{h}}/298\right)\text{e}^{\text{x}1}\right)/\left(1+\text{e}^{\text{x}2}\right)$ |  |
| --- | --- |

where

| $\begin{matrix} \text{x}_{1}=\Delta\text{H}*_{\text{A}}/\text{R}\left(\left(1/298\right)-\left(1/\text{T}_{\text{h}}\right)\right) \text{and} \\ \text{x}_{2}=\Delta\text{H}*_{\text{H}}/\text{R}\left(\left(1/\text{T}_{0.5\text{H}}\right)-\left(1/\text{T}_{\text{h}}\right)\right) \end{matrix}$ |  |
| --- | --- |

r(T_h_) represents the development rate (hr^−1^) at temperature T (°K) at hour h, p(25°C) is the development rate (hr^−1^) at 25°C assuming no temperature inactivation of the critical enzyme, ΔH*_A_ is the enthalpy of activation of the reaction that is catalyzed by the enzyme (cal/mol), ΔH*_H_ is the enthalpy change associated with high temperature inactivation of the enzyme (cal/mol), T0.5_H_ is the temperature (°K) where 50% of the enzyme is inactivated by high temperature, R is the universal gas constant (1.987 cal/mol/°C), and CD, represents cumulative development. The parameters were modified to match the EIP given in Focks et al. (2006), and to include a temperature of 40°C as a limit for mosquito survival (p_(25°)_ = 0.003; ΔH*_A_ = 13,000; ΔH*_H_ = 110,000; T_0.5H_ = 313) as shown in Figure A.

Figure A. Proportion of the EIP completed per day as a function of mean daily temperature.

Proportion of EIP

Mean daily temperature (°C)

As the daytime temperature range might affect development (Lambrechts et al., 2011), minimum and maximum temperature data was interpolated to obtain two hour intervals. A linear rise between 6 am and 2 pm (i.e. the time of minimum and maximum temperature, respectively) and a linear fall from 2 pm to 6 am of the following day was used. Thus r(T_h_) was calculated for 12 two hours’ intervals multiplied by 2 and added up.

References:

Focks D, Alexander N, Villegas E, Romero-Vivas C, Midega J, Bisset J, Morrison AC, Barrera R, Barbazan P, Sinh Nam V, Arredondo-Jiménez JI. 2006. Multicountry study of *Aedes aegypti* pupal productivity survey methodology: findings and recommendations. Geneva: TDR/IRM.

Jetten TH, Focks DA. 1997. Potential changes in the distribution of dengue transmission under climate warming. *American Journal of Tropical Medicine and Hygiene* 57: 285-297.

Lambrechts L, Paaijmans K, Fansiri T, Carrington L, Kramer L, Thomas M, Scott T. 2011. Impact of daily temperature fluctuations on dengue virus transmission by *Aedes aegypti*. *Proceedings of the National Academy of Science USA* 108:7460-7465.
