# Supplementary figures and images for: Is autumn the key for dengue epidemics in non endemic regions? The case of Argentina
Source: PeerJ. 2018 Jul 17;6:e5196. doi: 10.7717/peerj.5196 (PMC6054063; doi:10.7717/peerj.5196)

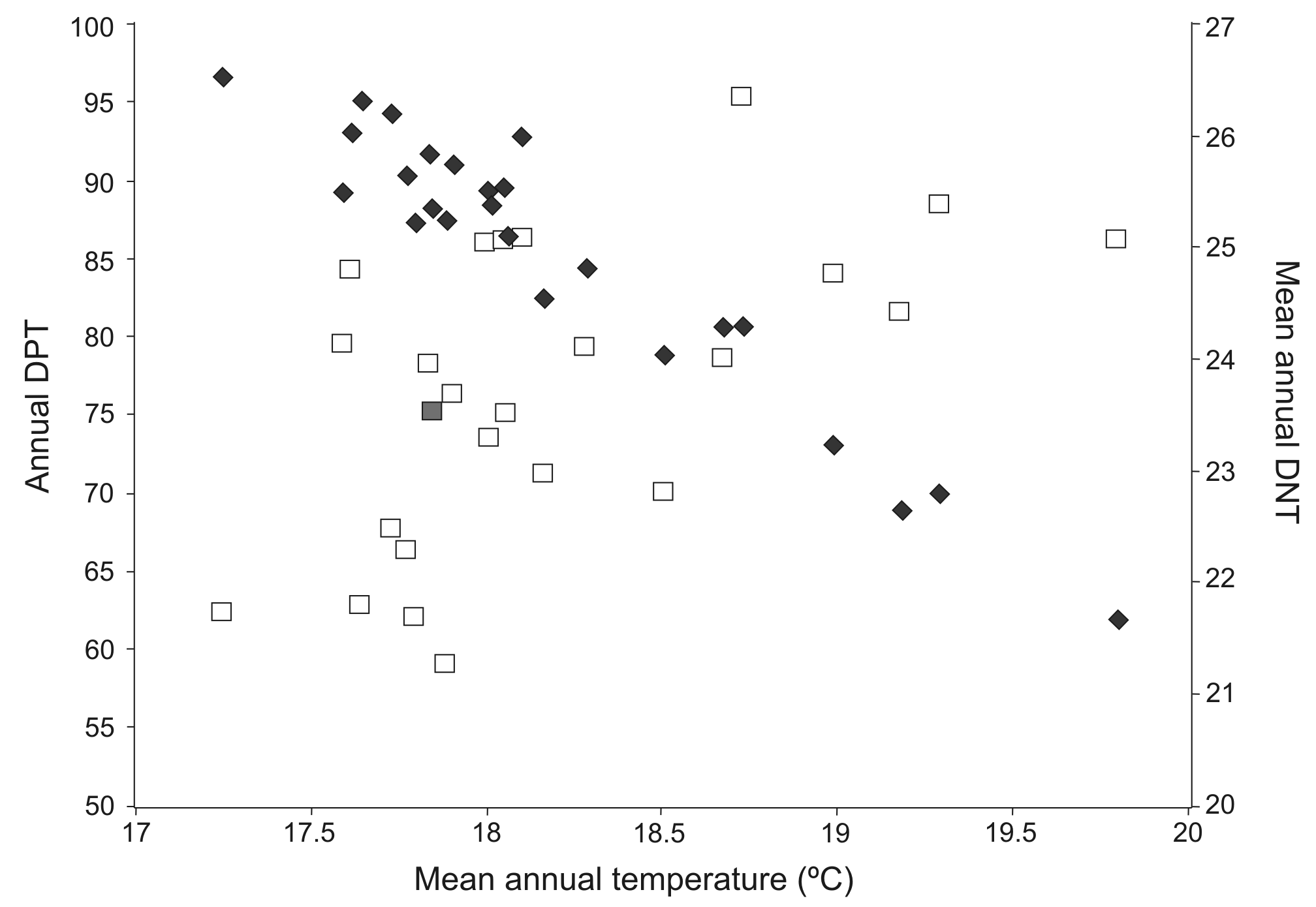

Supplement: File S3 — Each symbol represents a year between 1992 and 2016. [file peerj-06-5196-s003.png]
